# The Semantics of ‘Hip Pain’ and its Impact on Clinical Practice in Patient-Reported Outcome Measures (PROMs) of Disease: Results from a Clinical and Radiological Evaluation Cohort

**DOI:** 10.31138/mjr.31.4.389

**Published:** 2020-12-28

**Authors:** Mayuri Karela, Lloyd Rickard, Euthalia Roussou

**Affiliations:** 1 Barking Havering and Redbridge University Hospitals NHS Trust, Rheumatology and Rehabilitation, King George Hospital, Ilford, United Kingdom; 2 Barts and The London School of Medicine and Dentistry, London, United Kingdom

**Keywords:** Hip, pain, spondyloarthritis, PROMs

## Abstract

**Introduction:**

The item ‘hip pain’ is widely used in questionnaires related to Spondyloarthritis and/or Ankylosing spondylitis (AS), either in clinics with patients being physically present or remotely, as the hip joint is known to affect AS in particular. Patients in clinics often claim to have hip pain. However, by stating “hip” they are referring to variable structures located in the hip region not necessarily related to hip joint itself.

**Objective:**

To assess which structure(s) patients mean when referring to hip pain.

**Methods:**

A diagram used as a proforma for patients to indicate the site of ‘hip pain’ following a detailed history and examination was used. Radiological imaging was utilised for those patients with multiple sites or clinically unclear causes of “hip” pain.

**Results:**

From 54 patients 7 different anatomical sites described which were: Trochanter, (27.2%), hip joint (20.8%), iliac crests (anterior superior [6.9%], posterior superior [8.3%], and anterior inferior [4.1%]), lumbar spine (8.3%), sacroiliac joint (6.9%). More than 1 sites in the same patient: (17.5%). Diagnoses were: Trochanteric bursitis (27%), osteoarthritis of hip and spine, (25%), enthesitis (22%), sacroiliitis (6.7%), synovitis (5%), fibromyalgia (3.4%), and hip dislocation (1.6%). More than 1 diagnosis in same patient: 9.3%.

**Conclusion:**

’hip pain’ as an item used in questionnaires must be interpreted with caution.

## INTRODUCTION

Hip pain is incredibly common with an incidence of 19% in the general population aged over 65 years^1^, and 25% of all patients with joint-related pain.^2^

The underlying cause of hip pain can be challenging for clinicians to diagnose, due to its complex anatomy of bony, ligamentous, articular, and soft tissue structures.^2^ In addition, hip pain can be attributed to multiple disease processes, including inflammatory and degenerative arthritides, soft tissue disease, infection, and referred pain.^2^

The patients’ description of the location of the hip pain gives a strong indication as to the underlying diagnosis, with pain, felt anterior or in the groin indicating the actual articular hip joint in most cases.^2^ Despite the anatomical and diagnostic ambiguity behind the term ‘hip pain’, it is often used as a metric in Patient Assessed Measures (PAMs) of disease.^3,4^ PAMs are increasingly used within rheumatology as an important adjunct to traditional clinical, radiological and biochemical assessment.^5^ They allow for measurement of multi-dimensional outcomes including psychosocial well-being and physical functioning.^6^

The Assessment in Ankylosing Spondylitis Group (ASAS) recommends using PAMs in both research and clinical practice to assess domains including functional disability, spinal stiffness, and pain.^7^ Patient-reported ‘hip pain’ is also used to assess disease activity in the Bath Ankylosing Spondylitis Disease Activity Index (BASDAI),^3^ and has more lately been used as a self-reported diagnostic tool for Ankylosing Spondylitis (AS).^4^

Given the potential diagnostic and anatomical variability behind the term ‘hip pain’, this study investigated patients presenting to rheumatology clinic with ‘hip pain’. The aim was to: 1) assess the varying anatomical locations and disease processes underlying in the reported as ‘hip-pain’ complaint; and 2) evaluate the use of the term ‘hip pain’ in PAMs used to diagnose and assess disease activity in AS.

## METHODS

This was a prospective study registered as a scientific audit under the section “Clinical practice evaluation” of the audit proforma. As such, no consent was required.^8^ All patients (new and follow up) presenting with ‘hip pain’ during a one-year period (August 2014 to September 2015) age 18 and above were included. All patients were recruited by 3 independent clinicians aware of the study, in rheumatology clinics at King George’s Hospital, North East London (Essex).

**Figure 1.**
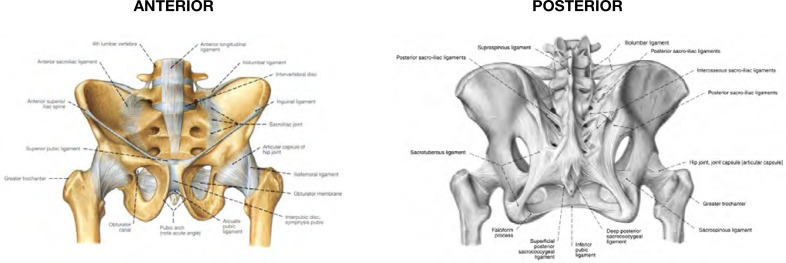
Audit proforma: Title of the audit “The semantics of hip pain”. Please complete in all patients presenting with the complaint of hip pain (to be completed by the attending clinician): Hospital number: Does the patient identify with having hip pain (do you have any pain in your hips?): Y / N Please indicate (x) on the diagram below where the patient experiences pain: Brief clinical description of location of pain:

After detailed history and examination performed on each patient, an accurate description of the site referred to as “hip” was recorded on a proforma designed for the purpose of the study (**Figure 1**). Schematic representation of the hip area produced by author LR. Radiological imaging was utilized, for those patients with multiple sites or clinically unclear causes of “hip” pain, to confirm or exclude the clinical diagnosis for the pain.

**Table:** 

Clinical impression/ diagnosis:	
Is English the patient’s first language:	Y / N
Is this Diagnosis supported by imaging studies (x-ray or u/s):	Y / N
Please return all completed Proformas to Dr xxxx (Project lead) for analysis.	Thank you.

Any cases where there were multiple sites of pain or the diagnosis was unclear had radiological imaging and laboratory investigations in order to confirm the diagnosis. This was part of routine clinical care and no additional tests were performed not related to standard clinical care.

The study has been registered as audit and did not go through the ethics committee in order to receive ethics board approval.

Data were collated in Microsoft Excel, and basic statistics including raw counts and percentages recorded.

## RESULTS

A total of 54 patients (M:F 10:44) were assessed with 14 of the 54 patients (25.9%) indicating more than one site for their hip pain, resulting in a total of 72 anatomical sites.

The Mean age for the cohort was 57.6 years (SD ±14.1). From the total of 54 patients, 40 patients (74%) indicated 1 site for the source of ‘hip pain’, while 14 indicated more than 1 site (10 patients 2 sites, [18.5%], 4 patients 3 sites [7.4%]). Radiological evaluation was required for 40/54 (74%). Out of those, 23 patients had X-rays (57.5%), 13 patients had ultrasound (32.5%), 4 patients (10%) had magnetic resonance imaging (MRI). In the remaining 14 patients, the diagnosis was clinical, and no radiological evaluation was required.

In total, 7 separate anatomical locations were indicated as the source of the complain of ‘hip pain’ shown in **Table 1**. The most common area was the trochanter (n=19; 26.3%). Pain related to the actual hip joint itself occurred in 20% of cases (n=15).

**Table 1.** Anatomical locality of reported hip pain.

**Anatomical location of Hip Pain**	**Number of patients N=54 (total sites 72)**	**Percentage of the incidence of each anatomical site referred to as hip pain by patients (%)**
Trochanter	19	27.2
Hip Joint	15	20.8
Lumbo-sacral Spine	6	8.3
Posterior Superior Iliac Crest	6	8.3
Anterior Superior Iliac Crest	5	6.9
Sacro-Iliac Joint	5	6.9
Anterior Inferior Iliac Crest	3	4.1

There was a wide range of diagnoses following the complaint/statement of ‘hip pain’ by the patients, with trochanteric bursitis being the most common (n=16; 30%) (**Table 2**). Hip pain that could be associated with inflammatory rheumatological causes was seen in less than half of cases (n=20; 37%). The second most common diagnosis was osteoarthritis (n=15; 28%).

**Table 2.** Diagnosis of reported ‘hip pain’ after investigation.

**Diagnosis**	Number of patients* N=54 (59 diagnoses)	**Percentage of the diagnoses from the 59 case notes obtained from 54 patients.**
Trochanteric bursitis	16	27.1
Osteoarthritis (Hips and / or spine)	15	25.4
Enthesitis	13	22
Sacroiliitis	4	6.7
Synovitis	3	5
Fibromyalgia	2	3.4
Hip dislocation	1	1.6

* 5 patients had more than 1 diagnosis.

## DISCUSSION

In this paper, the results of an audit related to the complaint stated by the patients as ‘hip pain’ are presented. The study was prospective, and results analysed retrospectively to define what patients mean by stating ‘hip pain’. The results suggest that when patients report ‘hip pain’, they can be referring to multiple anatomical locations and not necessarily the hip joint. Overall, there were 7 locations reported spanning much of the sacrum, pelvis, hip joint, surrounding soft tissue and bursae. This suggests a clear ambiguity of the semantics of the term ‘hip pain’ to a patient.

To medical professionals, when we refer to the hip, we mean the synovial ball and socket joint that articulates the femur with the acetabulum. In this study, however, only 20% of cases of ‘hip pain’ had an anatomical relationship to the hip joint itself.

This means that when we use PAM to score hip pain, there is clear ambiguity in the answers. Patients may be scoring pain that is not related to the hip joint at all.

Patients that already have a formal diagnosis of inflammatory arthritis may well be scoring pain associated with disease activity. However, the incidence of hip pain in patient self-reported groups is incredibly common,^1^ and patients are not precluded from having pain unrelated to their inflammatory arthritis. Scoring this hip pain may lessen the sensitivity and specificity of such PAMs such as the BASDAI. In addition, clinicians may, therefore, overestimate the degree of disease activity with potentially adverse clinical outcomes by prescribing unnecessary medications.

Only 37% of patients presenting to rheumatology clinic with ‘hip pain’ after referral had a diagnosis in keeping with inflammatory arthritis (n=20). It seems reasonable to presume that in the general population this incidence would be much lower.

Some PAMs^4^ use ‘hip pain’ to attempt to diagnose inflammatory arthritis through questionnaire remotely. We feel this should be utilized with caution as we have shown patient reported ‘hip pain’ to be diagnostically and anatomically varied. In addition, the majority of patient-reported ‘hip pain’ is not secondary to an inflammatory process, even in the rheumatology clinic.

Bias of the study may be related to the multi-ethnic population attending the hospital in which the audit has been carried out as stated before.^9,10^ To a proportion of patients, English is not their native language. To avoid confusion and with the aim to bypass potential language barriers, we used a diagram of the region. Patients were asked to draw on the diagram the exact site of pain.

## CONCLUSION

Self-reported ‘hip pain’ as a diagnostic aid for Ankylosing Spondylitis or Spondyloarthritis related to inflammatory back pain should be used with caution.

PAMs that use ‘hip pain’ to monitor disease activity are at risk of skewed results due to its heterogeneous diagnostic, anatomical & semantic nature.
